# Smartphone Apps Targeting Alcohol and Illicit Substance Use: Systematic Search in in Commercial App Stores and Critical Content Analysis

**DOI:** 10.2196/11831

**Published:** 2019-04-22

**Authors:** Babak Tofighi, Chemi Chemi, Jose Ruiz-Valcarcel, Paul Hein, Lu Hu

**Affiliations:** 1 Department of Population Health New York University School of Medicine New York, NY United States; 2 University of Puerto Rico School of Medicine San Juan Puerto Rico

**Keywords:** mHealth, substance use disorder, mobile health, alcohol abuse

## Abstract

**Background:**

Smartphone apps promise to enhance the reach of evidence-based interventions (cognitive behavior therapy, contingency management and therapeutic education system) for populations with substance use disorders, with minimal disruption to health systems. However, further studies are needed to systematically evaluate smartphone apps targeting alcohol and illicit substances.

**Objective:**

The aim of this study was to evaluate the functionality, aesthetics, and quality of information of free or low-cost apps claiming to target alcohol, benzodiazepine, cocaine, crack/cocaine, crystal methamphetamine, and heroin use using the validated Mobile App Rating Scale (MARS) and critical content analysis.

**Methods:**

A systematic search of iTunes and Google Play app stores for free or low-cost apps facilitating recovery was conducted in March 2018 and yielded 904 apps using the keywords described in previous studies (eg, recovery, sobriety, sober, alcohol, and heroin). An interdisciplinary team of clinicians, behavioral informatics, and public health reviewers trained in substance use disorders conducted a descriptive analysis of 74 apps categorized as reducing use. In addition to the MARS scale, a descriptive analysis of relevant apps was conducted by the study team to assess for quality indicators emphasized by expert guidelines and review articles.

**Results:**

Most apps (n=74) claimed to reduce use or promote abstinence and yielded an overall low median MARS score of 2.82 (0.55) and a wide range of scores (1.64, 4.20). Ratings were also low for engagement (2.75 (0.72)), functionality (3.64 (0.78)), aesthetics (3.03 (0.87)), information (2.82 (0.62)), and satisfaction (1.76 (0.67)) subdomains. Innovative design and content features elicited in the review included initial assessments of substance use following app download, tracking substance use, and related consequences (eg, cost or calorie intake), remote and proximate peer support per geospatial positioning, and allowing users and family members of individuals with substance use disorders to locate 12-step meetings, treatment programs, and mental health services. Few apps integrated evidence-based psychotherapeutic (eg, cognitive behavioral therapy [CBT] or motivational interviewing) and pharmacologic interventions (eg, naloxone or buprenorphine).

**Conclusions:**

Few commercially available apps yielded in our search integrated evidence-based interventions (eg, extended-release naltrexone, buprenorphine, naloxone, Self-Management and Recovery Training recovery, or CBT), and a concerning number of apps promoted harmful drinking and illicit substance use.

## Introduction

Mobile phone–based health (mHealth) interventions offer a ubiquitous and low-cost approach to improving health outcomes. Smartphone apps, short message service (SMS) text messaging, and interactive voice response are effective approaches to reducing the burden of substance use disorders (SUDs) [1-4]. Most Americans now own smartphones (77%), and smartphones are especially popular among younger adults aged 18 to 29 years (92%) [5]. Smartphone apps promise to enhance the reach of evidence-based interventions (cognitive behavior therapy, contingency management, and therapeutic education system) for populations with SUDs with minimal disruption to health systems [2,6-8]. Reports have estimated the availability of over 318,000 mHealth apps in 2017 and the use of health-related apps by approximately half of all smartphone users in 2018 [9]. Considering existing barriers to formal treatment for SUDs, including perceived stigma, cost, and limited treatment slots [10], smartphone apps are increasingly utilized by individuals excluded from care to reduce alcohol and illicit substance use [11-13].

Concerns regarding the quality, efficacy, and privacy of mHealth apps persist. App descriptions routinely include unsubstantiated claims of medical expertise and intervention efficacy, while failing to disclose the sale of health information and personal data gathered from the user to third party vendors for commercial use [14]. Studies assessing mHealth apps targeting SUDs in smartphone app stores (ie, Google Play and/or iTunes) mostly described commercially driven apps that failed to offer evidence-based psychosocial interventions or to link users to addiction treatment providers [11,12,15]. In a descriptive analysis of apps addressing alcohol use, Weaver et al also found that apps claiming to inform users of their possible blood alcohol concentration actually promoted risky drinking behavior via games and other entertaining features [12]. Searches also yielded many recreational apps that promoted drug cultivation, trafficking, and simulated use [16].

Despite these findings, further studies are needed to systematically evaluate smartphone apps targeting alcohol and illicit substances. Studies assessing app content are generally descriptive and limited to one-dimensional outcome quality measures [17]. Previous searches of apps targeting SUDs were often limited to a single app provider (iTunes or Google Play), did not use keywords described by users to search for apps targeting SUDs (ie, *recovery*, *sobriety*, *abstinence*, and *detox*), and did not incorporate validated methods of assessing smartphone apps [13,14,18]. In addition, in the last 3 years, as most of these searches were conducted, the number of mHealth apps has doubled from 165,000 apps in 2015 to 325,000 mHealth apps in 2017 [19].

Our study utilized the validated Mobile App Rating Scale (MARS)[18] and critical content analysis [14], which offered a standardized approach to evaluate the functionality, aesthetics, and quality of information of apps claiming to target alcohol, benzodiazepine, cocaine, crack/cocaine, crystal methamphetamine, and heroin use. The MARS is the first mHealth app–quality indicator of engagement, functionality, aesthetics, and informational content delivered by a multidisciplinary team of clinicians and technology experts [18]. Our research also utilized a critical content analysis of apps listed in the smartphone stores claiming to reduce substance use to assess actual clinical impact (ie, review of the literature and app developer website) and linkage to patient-centered care models for addiction treatment (ie, self-efficacy, education, linkage with addiction specialty care, primary care, self-help groups, and/or individual counseling) prioritized by public health experts [20,21].

## Methods

### Smartphone App Selection

**Figure 1:**
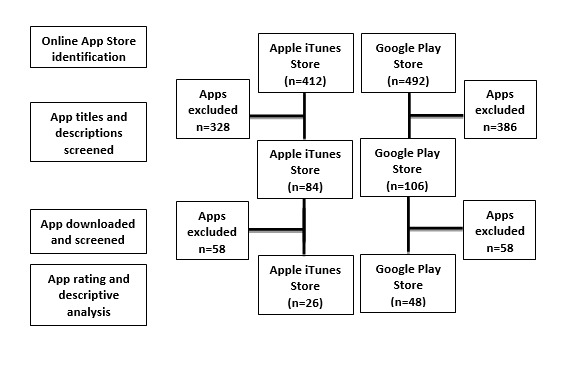
Flow diagram for smartphone app selection.

In March 2018, a systematic search of smartphone apps facilitating recovery from alcohol and illicit substances was conducted on the iTunes App Store and Google Play (see Figure 1) because of their popularity in the United States for app users. Additional apps targeting alcohol and illicit substance use described in the literature (eg, PubMed, Google Scholar, and PsycInfo) were also included in the search. The keyword search was in English, and apps that met the inclusion criteria were downloaded on an American mobile service network on the iPhone 6, iPhone 7, iPhone 7 plus, Samsung Galaxy J7, and Android LG G6 models.

The search terms were based on relevant terms described in the literature and in previous surveys among participants who utilized smartphone apps to reduce substance use (eg, *sober, sobriety, recovery, crystal methamphetamine, opioid, alcohol, cocaine, crack cocaine, and benzodiazepine*) [22]. The initial selection of apps excluded apps found to be irrelevant per the app title and online app store description (eg, music/relaxation, games, clocks, and religiosity), apps not in English, apps that lacked accessibility or functionality, apps designed for health care professionals, apps that cost more than $1 US dollars (on the grounds that they were unlikely to be purchased by a large number of users), and harmful apps promoting substance use. Although 12-step groups are an essential approach in reducing the burden of SUDs across diverse populations with SUDs [23], apps based solely on the 12-step model were not reviewed because of the large quantity of apps and limited study resources and their exclusion of content pertaining to medication-assisted treatments and effective behavior change models outlined by federal and expert guidelines [24]. The total number of apps yielded from our search was similar to previous studies on recovery apps [11,12,15]. Owing to the large number of apps retrieved in our initial search, apps without any user star ratings or reviews were also excluded in the preliminary screening. Apps that met preliminary inclusion criteria were then downloaded to the coauthors’ smartphones and assessed for accessibility, functionality, and relevancy of app content to reducing substance use (see Figure 1).

### App Assessment

App quality was assessed using the MARS [18]. The MARS domains (ie, engagement, functionality, aesthetics, information quality, and subjective quality) are constituted by 23 items based on a 5-point scale (1-Inadequate, 2-Poor, 3-Acceptable, 4-Good, and 5-Excellent). The classification domain is a descriptive survey of app price, platform, rating, and technical features (eg, password protection and log-in protocols). The excellent internal consistency (alpha=.92) and interrater reliability (intraclass correlation, ICC=.85) of the MARS scale make it ideal for conducting initial assessments of emerging apps [18]. Apps are then scored by calculating the mean scores of each respective subscale and the total mean score. The MARS scale has been successfully utilized to assess apps targeting a range of health conditions, including asthma, heart failure, and cancer [25-28]. However, the MARS scale has not been used to assess the quality of smartphone apps targeting alcohol and illicit substance use.

To ensure shared understanding of review criteria and MARS subscales, the study team reviewed the MARS literature and discussed each domain and subscale. The interdisciplinary study team included clinicians with experience in SUDs (BT and PH) and mHealth design (BT, PH, JRV, and LH). The reviewers convened and used 3 separate meetings to pilot apps and compare ratings per the MARS scale, assess the quality of their ratings, and resolve any discrepancies and ambiguities in the scale items. Each app was tested for at least 15 min. The raters then independently assessed 5 apps, and these apps were used to test the MARS tool, compare our judgments, and resolve any discrepancies. Interrater reliability among iTunes app raters was high (ICC=.80), but the overall app MARS score had fair interrater reliability (ICC=.58) requiring additional individual and group meetings to address discrepancies that emerged during the initial review. The study team compared ratings using iTunes for pragmatic reasons as most of the raters were Apple smartphone owners.

In the final round, we reviewed a total of 74 apps (26 Apple and 48 Google Play apps). Apps that met the inclusion criteria were assessed individually by the study authors on their smartphones in April 2018. In addition to the MARS scale, a descriptive analysis of relevant apps was conducted by the study team to assess for quality indicators emphasized by expert guidelines [8,29], as well as review articles [4,17,30], as evidence-based design practices in mHealth interventions targeting SUDs. This descriptive analysis elicited critical findings in app design and delivery features including (1) information or access to medication-assisted treatments via primary care and specialty addiction treatment programs (ie, residential treatment and intensive outpatient programs); (2) risk-reduction content (eg, safe sex practices, syringe exchange programs, naloxone, HIV, and hepatitis C virus [HCV] prevention education); (3) integration of behavior-change content in the intervention design or content (eg, cognitive behavioral therapy [CBT], motivational enhancement therapy, and contingency management); (4) empirical evidence demonstrating smartphone app efficacy by conducting searches in online databases (eg, PubMed and Google Scholar); and (5) privacy measures (eg, password protection, email or text verification, and user de-identification). Negative features of the reviewed apps assessed for the use of disruptive or distractive ads, religious texts, content exacerbating substance use (ie, access to discount liquor and bars with drink specials), and unverified claims of professional and clinical legitimacy. Finally, we identified apps that were aligned with the National Institute on Drug Abuse’s strategic objective of enhancing chronic disease management and personalized treatment that *matches an addicted person’s changing needs* over time based on the Medical Management outline (ie, patient-provider communication, medication adherence, self-management, goal of opioid abstinence, and counseling participation) to improve linkage of office-based opioid treatment with effective pharmacotherapies (ie, buprenorphine-naloxone and extended-release naltrexone) [31-34]. During the final round of reviews of apps that met the eligibility criteria, the primary author reviewed 30% of the apps individually assessed by the secondary authors to ensure consistency with the MARS subscales and descriptive analysis guidelines.

## Results

### Summary of Findings

The Google and iTunes searches identified a total of 904 apps of which 412 were on iTunes and 492 from Google Play (see Figure 1). The initial review excluded apps that were irrelevant (n=322), required payment for use (n=184), were linked with harmful use (eg, gamification of binge drinking or simulating illicit substance use; n=118), were duplicates (n=70), and were not in English (n=20).

Our secondary review then assessed a total 84 Apple apps and 106 Google apps that met the inclusion criteria. Apps were assessed individually by the study team, and 116 apps were excluded owing to the following factors: (1) unavailability in the app stores 3 weeks between the initial and secondary reviews (n=32); (2) duplicates (n=18); (3) educational content for health care providers (n=16); (4) required payments for full use of the app (n=9); (5) focused exclusively on the 12-step approach (n=9); (6) irrelevant to recovery (n=8); (7) not functioning (n=5); (8) required invitation from a treatment program (n=4); (9) required a device purchase (eg, breathalyzer; n=4); (10) only displayed a clock without any recovery content (n=4); (11) were not functional (n=3); (12) only offered religious content (n=2); (13) not in English (n=1); and (14) exacerbated harmful use (n=1).

Of the remaining apps, 74 met the inclusion criteria and underwent further analysis.

Most apps targeted alcohol use (n=40). Fewer apps emerged from this review exclusively addressing opioid use (n=6) and none focused on cocaine, crack/cocaine, or methamphetamine use. The overall median score of apps included in this study was poor (2.82 (0.55)) based on the 5-point MARS scale and demonstrated a wide range of ratings (1.64, 4.20; see Table 1). Scores for engagement (2.75 (0.72)), functionality (3.64 (0.78)), aesthetics (3.03 (0.87)), information (2.82 (0.62)), and satisfaction (1.76 (0.67)). Apps that had the highest average MARS score included SoberWorx (4.20), Recovery Today Magazine (3.77), Sober Grid (3.75), and Addicaid: Addiction Recovery and Support (3.64). The lowest-ranking apps included Sick Not Stupid (1.64), Sober Day Recovery App (1.71), and Stop Drinking Alcohol Now (1.75).

### Innovative Features of High-Quality Apps Targeting Substance Use Disorders

Use of the MARS scale and descriptive analysis allowed the study team to evaluate higher-rated apps (see Table 1) for design features that may be attributed to increased user engagement and potential clinical impact. Innovative design and content features elicited in the review included initial assessments, tracking substance use and related consequences (eg, cost and calorie intake), remote and proximate peer support per geospatial positioning, and allowing users and family members of individuals with SUDs to locate 12-step group meetings, treatment programs, and mental health services. Apps commonly elicited initial assessments of substance use patterns among users following app download. However, the only 2 apps that utilized an evidence-based approach to initial assessment were The Saying When and Alcohol Tracker apps.

The *Saying When* app (3.33) was developed by The Canadian Centre for Addiction and Mental Health to facilitate abstinence or reductions in the drinking quantity. The app features include an initial baseline assessment of drinking patterns, setting personalized goals, tracking drinks and urges, offering *tips for success*, and linkage to community treatment services. The self-help approach of the *Saying When* app was adapted from a manualized version based on *cognitive and behavioral strategies* that were not elaborated in the app store description and accompanying Web page. No scientific publications related to the app were available. However, app components appeared to be a cross-over of interventions described by the authors in earlier studies as having clinical impact in reducing drinking, including (1) exposure to initial assessments on drinking patterns [35] and (2) receipt of self-help books outlining initial assessments of drinking combined with the manualized version [36].

Alcohol Tracker (2.97) utilized the Alcohol Use Disorders Identification Test and Functional Analysis of Addictive Behaviors to notify users if they have surpassed alcohol consumption based on the National Institute for Health and Care Excellence UK Guidelines and link users to treatment resources. However, only users based in Singapore can access hotlines. In addition, reliance on self-reported drinking by apps tracking use is at risk of recall bias, particularly during binge-drinking episodes.

**Table 1:** Mobile app rating scale results. App availability is subject to removal per app developers, Google Play, and the Apple iTunes Store. Apps included in this table were rated as >3 or higher.

Smartphone app name	Engagement	Functionality	Aesthetics	Information	Satisfaction	Overall Score
SoberWorx	4.4	4.5	5	3.83	3.25	4.2
Recovery Today Magazine	3.8	4.5	4.3	3.5	2.75	3.77
Sober Grid	3.8	3.25	4.3	3.67	3.75	3.75
Addicaid: Addiction Recovery Support	3.8	4	4.67	3.5	2.25	3.64
BoozeFit	3.2	4.5	4	3.5	2	3.44
CleanTime Counter	2.2	4	4.25	3.5	3	3.39
Wise Drinking	3.2	3.5	4.667	2.83	2.75	3.39
SoberApp-Alcohol Calculator	3.4	4	3.33	3.67	2.5	3.38
Alcohol Check - BAC Calculator	3.4	3.5	4.3	3.4	2.25	3.37
FlexDek: Anglestrong Edition	3.8	4	3.3	3.16	2.5	3.35
Saying When	3.4	3.5	4	3.5	2.25	3.33
OARS Experience	2.8	3.75	2.5	4	3	3.21
Drinks Meter	2.75	4.25	2.67	3.5	2.75	3.18
Drive Sober	3.8	4.75	2.3	3.3	1.75	3.18
Best Alcohol Test	3.6	3.75	3.5	3	2	3.17
Stop OD NYC	2	4.25	3.3	3.28	3	3.17
Drug Addiction Recovery	2.6	3.75	4.3	3.6	1.5	3.15
Blood Alcohol Content Calculator+Timer	2.6	4.75	3.3	3	2	3.13
Alcohol Tracker	2.6	4.25	3.6	3.4	1.75	3.12
My Drink Control	3	4.25	3	3.6	1.75	3.12
AlcDroid Alcohol Tester	3	4.25	2.67	3.16	2.5	3.12
BACTrack	3.4	4.5	3.66	2.5	1.5	3.11
Sober Grid	3.75	3.3	3	3	2.5	3.11
Clean & Sober Time	3	3.5	4	3	2	3.1
Alcohol Calorie Counter	2.6	3.75	4.67	2.75	1.5	3.05
Intoxication Calculator	3.4	3.4	3.3	3.4	1.75	3.05
Addiction Quotes	3.2	4	3.3	3	1.75	3.05
Habit Tracker	2.6	4	3.3	3	2.25	3.03
Wbi.today	3.6	3.25	3.3	3.16	1.75	3.01

Several apps claimed to provide users access to sober peers via intra-app messaging, *help* icons, or forums. The most intriguing app (MARS score 3.75) facilitating peer support was *Sober Grid* and was developed by a team of academics and developers to offer a *global newsfeed* of shared posts on experiences in and insights into recovery and an instant help feature to link users to available peers online and in-person. The app then encourages patients to refer actively using peers to treatment. The app also encourages adoption among clinicians and health systems by offering an administrator dashboard, the option to launch mass notifications and messages to patients, and onboarding support. In addition, health systems may use the app to track substance use among patients during and post treatment and allow patients to meet and provide online support.

*Addicaid Recovery Support* (3.64) also offers linkage to peers in recovery and self-help group meetings. Some of the topics include *mothers in recovery, adult children of alcoholics, narcotics, methamphetamine, and heroin*. However, once a user joins the group, the posts are all from over a year ago. There are also a series of sessions such as *starting recovering*, *commitment and community*, *introducing a new routine*, and *recovery maintenance* and under each of these goals are comments including positive words of encouragement from peers. Once enabled, the app also provides users with a list of groups and meetings nearby.

*Pocket Rehab* facilitates text, telephone call, and video conferencing calls with other peers in recovery. Users are able to enter their location to search for nearby 12-step group meetings, a photo motivating sobriety, and an anticipated quit date. However, more interestingly, the app offers a *Community chat* forum with features similar to a Facebook wall and an immediate helpline linking individuals to other sober peers who are using the app and willing to communicate with the user to provide support. Users may select individual or community support and communicate via SMS text messaging, voice, or video. If this contact was not helpful, the app then links users to *chat with an experienced peer* or *write a journal entry*. Although the app promises to link individuals to other users within 1 to 2 min, our study team was unable to establish contact with any app user. The app requested access to users’ locations and assured that their information would not be available to third-party vendors. The study team gave a lower rating to the app (MARS score 2.66) owing to the lack of peer contact, linkage to behavioral health specialists in addition to peers in recovery, and integration of any evidence based-content.

Additional apps utilized fellow users to provide support. SoberWorx received a rating of 4.20 and was established by individuals in recovery to provide peer support, link to treatment resources, and also allow family members to locate treatment resources for loved ones with SUDs. Although the app claims to offer SMS text messaging–based contact with treatment programs via the app platform, we were unable to communicate with any providers or program staff. In addition to linking users to *treatment centers, addiction counseling, and sober living homes*, the app also allows for peer support, posting testimonials, and access to educational recovery content via YouTube videos. However, the peer support feature was also not interactive and was also difficult to identify online users and initiate contact.

The only app that clearly demonstrated application of an evidence-based psychotherapeutic approach was the Self-Management and Recovery Training (SMART )Recovery Cost Benefit Analysis (MARS rating 2.85). This app is based on the SMART recovery model, which integrates CBT to offer coping strategies for individuals in recovery to reduce the risk of relapse. The app allows users to enter the costs of ongoing use and the financial benefits of abstinence. However, the poor design (2) and lack of satisfaction (2) by the study team following app use resulted in a lower overall score. Despite the increasing popularity and clinical benefit of engaging with SMART recovery groups, counselors, and educational content based on the SMART platform [37], the app fails to effectively translate these resources into accessible and user-tailored features.

### Government-Sponsored Smartphone Apps

Our search yielded few government-initiated apps and apps that offered risk reduction measures for individuals with SUDs. The most intriguing app was STOP OD NYC (3.17), developed by the New York City Department of Health and Mental Hygiene, which provides detailed information on opioids (eg, heroin and fentanyl) and instructions on naloxone administration in the event of an overdose. The app provides several risk-reduction content, including (1) a *find naloxone* option that links users to mapped pharmacies, harm reduction programs, and health care centers providing free naloxone; (2) naloxone administration instructions for intramuscular, intranasal, and auto-injector formulations of naloxone; (3) recognizing individuals suspected of an overdose; and (4) information on legal protection for individuals administering naloxone. The app utilizes SMS text messaging, cartoon, and YouTube-based videos to offer users multimedia educational instructions. Finally, users can click on the *NYC Health* icon to access other health resources within the Department of Health and Mental Hygiene platform (eg, cardiovascular health, reducing glucose intake, and smoking cessation).

*My Drink Control* (3.12) is an app and Web-based tool developed by the Public Health Department in Zurich, Switzerland, to facilitate tracking of alcohol use, financial costs of use, and calories gained with each drink. Although the app’s *psychoeducational* content was based on CBT, our review of the app failed to elicit any theory-based content or design features. The app’s overall functionality was limited to tracking drinks, reminders, and linkage to treatment and counseling services for alcohol use. However, compared with other government-sponsored apps, *My Drink Control* had a more appealing and user-friendly design.

Furthermore, 1 app designed for the Substance Abuse and Mental Health Services Administration (SAMHSA) app challenge, *FlexDek MAT* (2.90), described itself as linking participants to the methadone maintenance program but again failed to offer accurate and updated contact information regarding the Office based opioid treatment (OBOT) programs after this feature was utilized. Information pertaining to MAT, including *buprenorphine*, *methadone*, *MAT*, *naltrexone*, and *after naltrexone*, was limited to PDF files, links to SAMHSA’s website, and an external website [38]. Other links to the 12-step and SMART recovery groups were not functioning after linking to an external website. The *forum* icon opened an error page and the *Coaches* option linked users to only 5 recovery coaches across the nation with a nonfunctioning link icon. The *rewards* option for using the app claimed to offer free hours of recovery coaching, but this was not evident in our review. In addition to linking to nonfunctioning pages, the app also exposed users to irrelevant ads in the lower segment of the screen.

### Features of Low-Quality Apps Targeting Substance Use Disorders

Lower quality apps often claimed to support recovery through complex design features but were limited to 1 to 2 basic functions such as supportive quotes, timers, blood alcohol calculators, or logging daily substance use without providing tailored feedback and evidence-based interventions. Some apps only copied quotes from religious texts without specifying content in the title or description (eg, OARS experience). Furthermore, 1 app entitled *Alcoholism Treatment* would only play ambient electronic music that app developers claimed would stimulate desires to quit.

Many of these apps used deceptive descriptions of complex recovery resources but would inundate users with pop-up advertisements (eg, Stop Drinking Alcohol Now and Sobriety Clock), require users to log-in via Facebook or Google with access to their social networks, and request access to user location. Among blood alcohol calculators, most lacked useful information on evidence-based treatment approaches and were often rated poorly by our study team and app store users. In addition, 1 app, BACtrack, developed by the San Francisco–based BACtrack, enables users to measure blood alcohol levels. However, to utilize the app, users were required to purchase a breathalyzer ($99.00 US dollars) to fully utilize the platform. Users were instructed to breathe into the breathalyzer and promised to log results wirelessly to their mobile device. The only free and functioning feature on the app was a link to Uber to access a ride. Additional apps also claimed to have initiated supportive peer networks, but forums and peer-messaging functions would typically be inactive. Others would link users to 12-step group meeting schedules or 12-step–based online forums rather than the app’s own support networks. Motivational content would typically appear in the form of quotes without any integration of behavior-change principles (eg, Recovery Quotes, Clean & Sober Recovery, and Addiction Quotes).

Other features of low-quality apps included the use of basic functions such as motivational quotes, timers, or informational content to ultimately expose users to pop-up advertisements for a single private residential treatment program or clinician (eg, SoberBud, Sobriety Clock, and Stop Drinking Now). Other apps would solicit users to pay for the *full-version* app to receive more comprehensive recovery content (eg, Hypnosis for Alcoholism, Addiction and Recovery, and Sober Tree).

Some apps were concerning for the possibility of exacerbating substance use. The *Drugs* app offered informational content that was not easily accessible, and the forum included threads with individuals offering to sell drugs. The *Drive After: Alcohol calculator* app offered users information on strategies to *feel more sober* or conceal the odor of alcohol. *Best Alcohol Test* is intended to offer a blood alcohol level calculator but also offers users games to test their reflexes following binge-drinking episodes.

Another app described as *Best Home Cure for Alcoholism* (2.38) claimed to offer alternative remedies for alcohol use and withdrawal symptoms with topics such as *how to make someone stop drinking alcohol forever*; *how to quit alcohol ayurvedic*; and *how to stop alcohol drinking of my husband*. However, content was limited to increasing intake of fruits, vegetables, juices, water, and coffee without offering any empirical evidence on these approaches or elaborating on the credibility of the article authors and app designers.

## Discussion

### Summary

The initial search for apps targeting substance use yielded 74 apps; however, only 7 apps offered any evidence-based content, such as information on effective pharmacotherapies for SUDs (n=3), harm-reduction content (n=1), or behavior-change principles within app content or design features (n=3). None of the apps facilitated linkage to primary care–based treatment for SUDs, methadone maintenance treatment programs for Opioid use disorder (OUD), or clarified insurance requirements or costs related to available primary care or specialty addiction treatment programs.

Although none of the apps cited any empirical evidence suggesting potential clinical benefit or sustained engagement among users, app quality assessment via the MARS score offers a useful approach before evaluating for efficacy. The apps in this study had a low overall median quality MARS score of 2.81. However, the overall low information (2.81), engagement (2.75), and satisfaction (1.75) subscale scores highlight the lack of evidence-based content and gap in intervention design.

Although numerous apps emerged from the review targeting alcohol use, only STOP OD NYC and FlexDek MAT specifically targeted opioid use with evidence-based content (eg, effective pharmacotherapies for OUD). However, STOP OD NYC was limited to naloxone and overdose prevention and FlexDek MAT lacked basic functionality, was not aesthetically engaging, and the informational content was limited to PDF files and external links to the SAMHSA Web page. None of the commercially developed apps offered any informational content or access to online or clinical resources addressing harm-reduction practices and HIV and HCV prevention or screening content.

Few apps integrated evidence-based behavior change content (eg, SMART Recovery Cost Benefit) and mostly centered on basic informational content summarizing how many calories or money would be saved with alcohol abstinence, tracking time of abstinence with timers, using graphs to chart quantities of consumed alcoholic beverages, and basic information about addiction (eg, as a chronic disease), improvised *tips* on recovery, or quotes from the Bible that were not aligned with evidence-based psychotherapeutic approaches.

### Empirical Evidence Demonstrating Smartphone App Efficacy

Although smartphone software apps are technologically capable to enhance care for SUDs with complex and multifaceted interventions, our review found that even apps with higher MARS ratings were typically limited to singular functions (eg, validated assessments such as Alcohol Use Disorders Identification Test (AUDIT) or Alcohol, Smoking and Substance Involvement Screening Test (ASSIST), active peer support systems, access to 12-step or SMART recovery group meetings, and/or linkage to specialty addiction treatment providers).

In a similar review of alcohol-related smartphone apps in 2012 by Weaver et al, only 44 of the 500 apps that met the initial inclusion criteria actually targeted reductions in alcohol use, and none presented evidence of efficacy [12]. Despite a doubling in apps targeting alcohol use in a subsequent review in 2014, there was no evidence of improved integration of evidence-based approaches (eg, effective psychotherapeutic interventions) and most promoted alcohol use [39]. Our review of commercially available alcohol-reduction apps is aligned with previous findings limiting their use in real-world clinical settings and is concerning for exacerbating relapse or worsening alcohol use [12,32].

MARS ratings of commercially available apps in this study also parallel the critical analysis of apps targeting heart failure and mindfulness and their lack of evidence-guided content [28,40]. These findings contrast with randomized controlled trials demonstrating effectiveness for university-developed smartphone apps targeting alcohol use [2,41] and cravings [7,42]. However, our review found that these apps remain unavailable for individual use via existing app stores and without invitation by a licensed addiction treatment provider.

Although the apps lacked any clinical evidence of efficacy, there was also no evidence of sustained use. For instance, apps claiming to offer peer support via forums or SMS text messaging contact with other users were not active or unresponsive. In addition, forums lacked moderators or clinicians who could offer evidence-based responses to forum threads. Long-term engagement with technology-based interventions is critical to ensure behavior change and meaningful clinical outcomes. Challenges to larger-scale adoption of mHealth interventions include the lack of open mHealth frameworks that clarify underlying mechanisms linking intervention design features, effective psychotherapeutic approaches, and clinical outcomes. For instance, even basic process measures, such as the duration or intensity of app utilization, are not assessed or disclosed. Instead, users and researchers alike must rely on other user ratings and comments to gauge an app’s potential benefit. Thus, preliminary studies are needed among participants in the community or addiction treatment settings assessing the impact of self-reported app usage targeting substance use and treatment utilization. Future smartphone app research requires elucidation of how app design features, content, and behavior-change principles impact targeted clinical outcomes. In July 2017, the Food and Drug Administration (FDA) launched the Digital Health Innovation Action Plan to offer additional oversight over clinical and patient decision support software. Further oversight by the FDA may facilitate transparency in app design, promotion of clinical studies assessing app safety and effectiveness, and increase the confidence of users and health systems for broader adoption [43].

### Harm Reduction or Exacerbating Harmful Use?

Apps addressing alcohol use linked participants to app-based transportation companies or sober peers to facilitate rides if they were intoxicated. Some of the apps offering blood alcohol calculators for alcohol encouraged users to avoid reaching intoxication or hazardous drinking levels. However, other blood alcohol calculator apps would use cartoon imagery to gamify drinking and even allow users to compare their blood alcohol levels with other drinking peers. Another blood alcohol calculator app would offer users information about how to hide odors of alcohol in their breath or *sober up* if they exceeded certain levels of drinking. Our findings are aligned with reviews of smartphone apps in the last decade, emphasizing the availability of commercially developed apps that mostly exacerbate rather than mitigate harmful substance use [12,16,39]. Studies have reported the dramatic rise of apps promoting cigarette smoking [44], cannabis [15], and alcohol use [12,39]. In 2012, Bindhim et al searched for *cannabis, weed, marijuana, cocaine, heroin, and ecstasy* and reported an increase in harmful apps from 238 apps in February 2012 to 410 apps in May 2012 that encouraged contact with actively using peers, role-playing as cartel bosses or cannabis farmers, or simulating substance use [16]. Not surprisingly, in 2017, Google blocked approximately 700,000 of the nearly 3.5 million Android apps purged for promoting violence, hate, adult material, illicit activities, and substance use [45]. However, with 200 new apps entering the marketplace daily [19], app stores must develop more stringent restrictions considering the consistent growth in apps encouraging harmful substance use.

### Linkage to Treatment

Although numerous descriptions attracted potential users to the app’s capacity to link them to nearby treatment programs, nearly all lacked updated information about available programs, were not tailored to uninsured or Medicaid-insured patients, or would direct users to a single private practice therapist or residential treatment program even if the investigators were attempting to request an office-based opioid treatment program. The deceptive referral of all requests for treatment to a commercial advertisement or to a single private practice was common. Among apps developed by government agencies, users were able to eventually locate treatment programs after being redirected to government Web pages that listed available clinics but were not adapted for mobile phone–based internet browsers (eg, STOP OD NYC and Drive Sober Alabama). However, the STOP OD NYC app’s overall design is the most ideally suited platform to integrate harm-reduction resources (ie, syringe exchange programs and naloxone) as well as linking users to low-cost office-based opioid treatment programs for buprenorphine and extended-release naltrexone treatment and the high density of 12-step and SMART recovery groups within New York City.

### Integration of Behavior-Change Content

CBT and relapse prevention strategies offer a collaborative, individualized, psychological treatment recognized as effective approaches to generating behavioral, cognitive, and emotional adaption to a wide range of common psychological problems [46]. The efficacy of CBT and relapse prevention strategies has been supported by a comprehensive review of 106 meta-analyses across different clinical groups that also extends to SUDs. Despite their widespread adoption in academically developed smartphone apps (eg, A-CHESS), only the SMART cost-benefit app utilized behavior change models in this review.

SMART Recovery is based on both the Rational-Emotive-Behavior therapy and CBT approaches and reinforces learning skills to cope with (rather than avoid) emotional disturbances that exacerbate substance use [37,46]. The SMART Recovery website offers extensive resources, including articles, podcasts, videos, and self-help assignments that deepen user engagement with this approach. In addition, its online forum is active and offers unique discussion threads, including *Building and Maintaining Motivation, Coping with Urges, Managing Thoughts, Feelings, and Behaviors, Living a Balanced Life*, and specialized peer support group forums based on specific substances and a *family and friends* forum. The Smart Cost Benefit app has tremendous potential to integrate an already vibrant online forum and evidence-based psychotherapeutic approach.

Participation in self-help support groups, online forums, and even smartphone app communities can help motivate users to engage in healthy activities. A supportive app community can help users share and discuss their recovery experiences and the challenges of regular practice. This could potentially complement or substitute for the support provided in face-to-face recovery treatment modalities. Although nearly many of the reviewed apps provided social network–sharing, few offered moderators or clinicians to guide discussions. Further research is needed to assess the impact of app-based forums and peer SMS text messaging to enhance engagement with the app and clinical outcomes.

### Privacy Measures

Apps generally lacked the use of privacy measures to protect health information: few required password protection, elucidated the use of security certifications from cellphone providers, utilized encryption technologies for transmitted content, or 2-step verification during registration. Numerous apps asked participants for access to their Facebook profile and/or Google profile during registration and reassured users in the Terms and Conditions that their personal information would be safeguarded. Several apps were also used for research purposes but did not specify details of the research study, rights as a study subject, and how to terminate one’s participation in the study and remove their data usage information. The Health Insurance Portability and Accountability Act and expert guidelines have outlined several measures to ensure the privacy of patient-physician communication in emerging health information technologies, including (1) the use of simple message content that refrains from disclosing patient name, diagnosis, or enrollment in treatment; (2) encouraging 2-step verification, password protection, and finger Touch identification; (3) obtaining security certifications from cellphone providers; (4) using encryption technologies for user responses; and (5) regularly deleting or setting expiration periods for communication content [47].

### Limitations

This is one of the first comprehensive studies to review apps targeting illicit substances using the MARS scoring criteria and provide a reliable measure of engagement, functionality, visual appeal, and informational quality. Furthermore, this is the only review to assess for the integration of evidence-based psychotherapeutic or pharmacological approaches to SUDs and their applicability to office-based management of SUDs based on the medical management model. However, findings emerging from our descriptive analysis are not based on validated usability and/or efficacy study methods and require more rigorous study methods to assess the clinical impact. The review was limited to Google Play and Apple iOS and did not include apps available in F-Droid, Amazon Appstore, and GetJar, among other smaller scale app platforms. Our assessment of user privacy did not incorporate open-source developer codes for malicious purposes.

### Conclusions

Online app stores offer unprecedented opportunities to expand access to effective harm reduction and treatment approaches for individuals with SUDs. However, the overall low MARS scale ratings and findings emerging from our descriptive analysis highlight the lack of evidence-based apps for individuals seeking additional support. Further studies are needed to assess the impact of existing evidence-based apps described in this review (eg, STOP OD NYC and FlexDek MAT). Investigators should leverage online app stores to assess the acceptability and clinical impact of effective apps targeting SUDs that are not yet available within online app stores for individual use (eg, A-CHESS). Finally, public health experts should utilize the popularity of online app stores to offer user-friendly and evidence-based apps that facilitate access to effective pharmacotherapies for SUDs, harm-reduction resources (eg, naloxone and syringe exchange programs), specialty addiction treatment programs (eg, intensive outpatient programs and methadone maintenance programs), and linkage to primary care–based treatment for SUDs.

## Abbreviations

ASSIST: Alcohol, Smoking and Substance Involvement Screening Test
AUDIT: Alcohol Use Disorders Identification Test
CBT: Cognitive Behavioral Therapy
FDA: Food and Drug Administration
HCV: hepatitis C virus
ICC: intraclass correlation
MARS: Mobile App Rating Scale
mHealth: mobile health
OBOT: Office based opioid treatment
OUD: Opioid use disorder
SAMHSA: Substance Abuse and Mental Health Services Administration
SMART: Self-Management and Recovery Training
SMS: short message service
SUD: substance use disorder
